# 1-[5-Methyl-1-(4-nitro­phen­yl)-1*H*-1,2,3-triazol-4-yl]ethanone

**DOI:** 10.1107/S1600536813029425

**Published:** 2013-10-31

**Authors:** N. Vinutha, S. Madan Kumar, Nithinchandra, Kalluraya Balakrishna, N. K. Lokanath, D Revannasiddaiah

**Affiliations:** a Department of Studies in Physics, University of Mysore, Manasagangotri, Mysore 570 006, India; b Department of Studies in Chemistry, Mangalore University, Mangalagangotri, Mangalore 574 199, India

## Abstract

The asymmetric unit of the title compound, C_11_H_10_N_4_O_3_, contains two independent mol­ecules in which the benzene rings make dihedral angles of 38.3 (2) and 87.1 (2)° with respect to the triazole rings. In the crystal, the mol­ecules are linked by C—H⋯O hydrogen bonds, forming chains along [021]. Further, weak C—O⋯π [3.865 (5) Å, 83.8 (3)°] and N—O⋯π [3.275 (5) and 3.240 (6) Å, 141.8 (4) and 102.8 (3)°] inter­actions are observed.

## Related literature

For chemical and biological properties and pharmocological applications of 1,2,3-triazole derivative, see: Nithinchandra *et al.* (2012 ▶, 2013 ▶); Biagi *et al.* (2004 ▶); Manfredini *et al.* (2000 ▶); Sherement *et al.* (2004 ▶). For bond-length data, see: Allen *et al.* (1987 ▶).
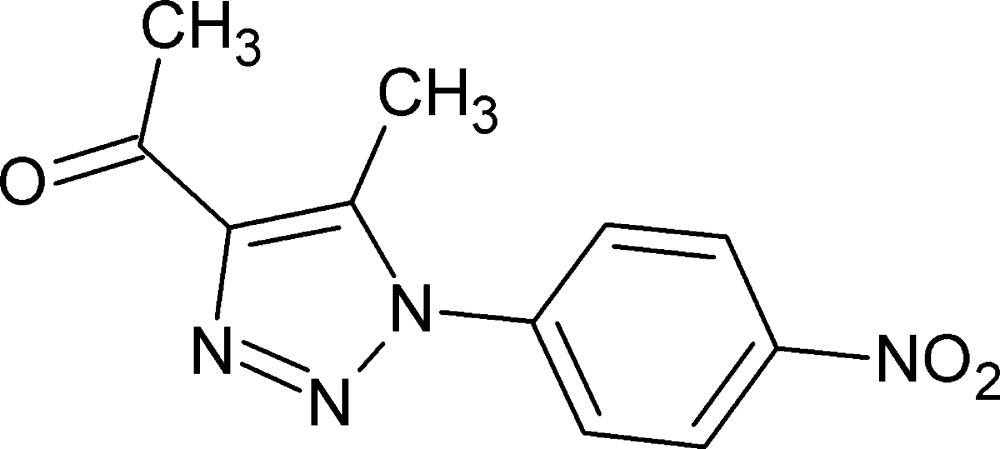


## Experimental

### 

#### Crystal data

C_11_H_10_N_4_O_3_
*M* *_r_* = 246.23
Orthorhombic, 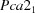

*a* = 7.2786 (10) Å
*b* = 11.5055 (16) Å
*c* = 27.220 (4) Å
*V* = 2279.5 (6) Å^3^
*Z* = 8
Cu *K*α radiation
μ = 0.91 mm^−1^
*T* = 296 K
0.23 × 0.22 × 0.21 mm

#### Data collection

Bruker X8 Proteum diffractometer
Absorption correction: multi-scan (*SADABS*; Bruker, 2013 ▶) *T*_min_ = 0.818, *T*_max_ = 0.831
8697 measured reflections
1910 independent reflections
1617 reflections with *I* > 2σ(*I*)
*R*_int_ = 0.057

#### Refinement

*R*[*F* ^2^ > 2σ(*F* ^2^)] = 0.046
*wR*(*F* ^2^) = 0.138
*S* = 1.08
1910 reflections
329 parameters
1 restraint
H-atom parameters constrained
Δρ_max_ = 0.17 e Å^−3^
Δρ_min_ = −0.17 e Å^−3^

### 

Data collection: *APEX2* (Bruker, 2013 ▶); cell refinement: *SAINT* (Bruker, 2013 ▶); data reduction: *SAINT*; program(s) used to solve structure: *SHELXS97* (Sheldrick, 2008 ▶); program(s) used to refine structure: *SHELXL97* (Sheldrick, 2008 ▶); molecular graphics: *Mercury* (Macrae *et al.*, 2008 ▶); software used to prepare material for publication: *Mercury*.

## Supplementary Material

Crystal structure: contains datablock(s) global, I. DOI: 10.1107/S1600536813029425/is5313sup1.cif

Structure factors: contains datablock(s) I. DOI: 10.1107/S1600536813029425/is5313Isup2.hkl

Additional supplementary materials:  crystallographic information; 3D view; checkCIF report

## Figures and Tables

**Table 1:** Hydrogen-bond geometry (Å, °)

*D*—H⋯*A*	*D*—H	H⋯*A*	*D*⋯*A*	*D*—H⋯*A*
C5*B*—H5*B*1⋯O3*A* ^i^	0.96	2.51	3.306 (8)	141
C10*B*—H10*B*⋯O1*A*	0.93	2.58	3.197 (7)	124

Symmetry code: (i) 
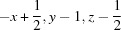
.

**Fig. 1.**
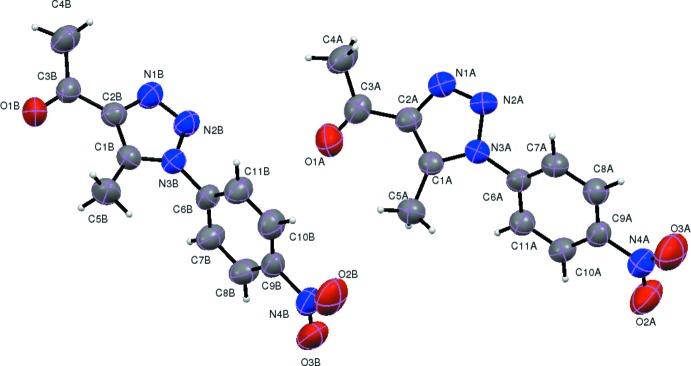
Asymmetric unit of the title compound with 50% probability ellipsoids.

**Fig. 2.**
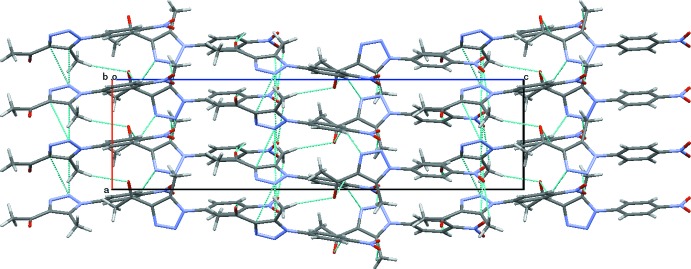
Packing diagram of the title compound, viewed along the b axis. Dotted lines indicate hydrogen bonds and short contacts involved.

## supplementary crystallographic information

### 1. Comment

1,2,3-Triazoles are attractive constructs, because of their unique chemical properties and they find many applications in organic and medicinal chemistry (Nithinchandra *et al.*, 2013). They are found to be potent antimicrobial (Sherement *et al.*, 2004)and antiviral agents. Some of them have exhibited antiproliferative and anti-inflammatory property (Nithinchandra *et al.*, 2012). Also, 1,2,3-triazoles are used as DNA cleaving agents (Manfredini *et al.*, 2000) and potassium channel activators (Biagi *et al.*, 2004).

The asymmetric unit of of the title compound consists of two molecules *A* and *B* (Fig. 1). They show conformational difference, as evident from dihedral angles. The dihedral angle between benzene ring and triazole moiety is 38.2 (3)° in *A* and 87.6 (4)° in *B*. The values of the bond lengths are similar to the reported literature (Allen *et al.*, 1987).

In the crystal, the molecules are linked to one another with the C—H···O hydrogen bonds (Table 1). Also, short contacts of the type C3A—O1A···*Cg*4(*x* + 1/2, *y* - 1, *z*) with a distance 3.865 (5) Å [angle 83.8 (3)°], N4A—O2A···*Cg*3(*x* - 1, *y* - 1, *z* + 1/2) with a distance 3.275 (5) Å [angle 141.8 (4)°] and N4B—O3B···*Cg*1(*x* - 1/2, *y* - 1, *z*) with a distance 3.240 (6) Å [angle 102.8 (3)°] help in crystal stabilization. These interactions form a three dimensional architecture (Fig. 2), where *Cg*1, *Cg*3 and *Cg*4 are the centroids of the N1A/N2A/N3A/C1A/C2A, N1B/N2B/N3B/C1B/C2B and C6B–C11B rings, respectively.

### 2. Experimental

1-Azido-4-nitrobenzene (0.01 mol) was treated with acetyl acetone (0.01 mol) in methanol (10 ml) and the mixture was cooled to 0 °C. Sodium methoxide (0.01 mol) was added under inert atmosphere to the above mixture and stirred at ambient temperature for 8 h. Progress of the reaction was monitored by TLC (ethyl acetate/petroleum ether, 2:3, *v*/*v*). After completion of the reaction, the mixture was poured onto ice cold water. The precipitated solid was filtered, washed with water and recrystallized from ethanol. Single crystals suitable for X-ray analysis were obtained from a 1:2 mixture of DMF and ethanol by slow evaporation.

### 3. Refinement

All the H atoms were fixed geometrically (C—H = 0.93–0.96 Å) and allowed to ride on their parent atoms with *U*_iso_(H) = 1.5*U*_eq_(*C*-methyl) and 1.2*U*_eq_(C) for other H atoms. The Flack parameter *x* refines to 0.2 (4) with unmerged data, and the absolute structure cannot be determined reliably. The final refinement was performed with the merged data.

### Crystal data

**Table:**

C_11_H_10_N_4_O_3_	*F*(000) = 1024
*M**_r_* = 246.23	*D*_x_ = 1.435 Mg m^−^^3^
Orthorhombic, *P**c**a*2_1_	Cu *K*α radiation, λ = 1.54178 Å
Hall symbol: P 2c -2ac	Cell parameters from 1910 reflections
*a* = 7.2786 (10) Å	θ = 3.2–64.2°
*b* = 11.5055 (16) Å	µ = 0.91 mm^−^^1^
*c* = 27.220 (4) Å	*T* = 296 K
*V* = 2279.5 (6) Å^3^	Block, red
*Z* = 8	0.23 × 0.22 × 0.21 mm

### Data collection

**Table:**

Bruker X8 Proteum diffractometer	1910 independent reflections
Radiation source: Bruker MicroStar microfocus rotating anode	1617 reflections with *I* > 2σ(*I*)
Helios multilayer optics monochromator	*R*_int_ = 0.057
Detector resolution: 10.7 pixels mm^-1^	θ_max_ = 64.2°, θ_min_ = 3.3°
φ and ω scans	*h* = −8→8
Absorption correction: multi-scan (*SADABS*; Bruker, 2013)	*k* = −5→13
*T*_min_ = 0.818, *T*_max_ = 0.831	*l* = −30→31
8697 measured reflections	

### Refinement

**Table:**

Refinement on *F*^2^	Primary atom site location: structure-invariant direct methods
Least-squares matrix: full	Secondary atom site location: difference Fourier map
*R*[*F*^2^ > 2σ(*F*^2^)] = 0.046	Hydrogen site location: inferred from neighbouring sites
*wR*(*F*^2^) = 0.138	H-atom parameters constrained
*S* = 1.08	*w* = 1/[σ^2^(*F*_o_^2^) + (0.0932*P*)^2^] where *P* = (*F*_o_^2^ + 2*F*_c_^2^)/3
1910 reflections	(Δ/σ)_max_ < 0.001
329 parameters	Δρ_max_ = 0.17 e Å^−^^3^
1 restraint	Δρ_min_ = −0.17 e Å^−^^3^

### Special details

**Table:**

Geometry. Bond distances, angles *etc*. have been calculated using the rounded fractional coordinates. All su's are estimated from the variances of the (full) variance-covariance matrix. The cell e.s.d.'s are taken into account in the estimation of distances, angles and torsion angles
Refinement. Refinement on *F*^2^ for ALL reflections except those flagged by the user for potential systematic errors. Weighted *R*-factors *wR* and all goodnesses of fit *S* are based on *F*^2^, conventional *R*-factors *R* are based on *F*, with *F* set to zero for negative *F*^2^. The observed criterion of *F*^2^ > σ(*F*^2^) is used only for calculating -*R*-factor-obs *etc*. and is not relevant to the choice of reflections for refinement. *R*-factors based on *F*^2^ are statistically about twice as large as those based on *F*, and *R*-factors based on ALL data will be even larger.

### Fractional atomic coordinates and isotropic or equivalent isotropic displacement parameters (Å^2^)

**Table:**

	*x*	*y*	*z*	*U*_iso_*/*U*_eq_	
O1A	0.1681 (6)	0.7317 (4)	0.30010 (16)	0.0907 (16)	
O2A	0.5412 (8)	0.8545 (4)	0.64933 (16)	0.1063 (18)	
O3A	0.4670 (10)	1.0325 (5)	0.64815 (18)	0.129 (3)	
N1A	0.4033 (6)	0.9759 (4)	0.35049 (15)	0.0680 (16)	
N2A	0.4450 (6)	0.9990 (4)	0.39568 (14)	0.0657 (14)	
N3A	0.3707 (5)	0.9122 (3)	0.42410 (12)	0.0522 (11)	
N4A	0.4913 (7)	0.9402 (4)	0.62761 (15)	0.0767 (16)	
C1A	0.2812 (6)	0.8331 (4)	0.39621 (15)	0.0497 (12)	
C2A	0.3036 (6)	0.8752 (4)	0.34871 (16)	0.0570 (14)	
C3A	0.2316 (7)	0.8288 (5)	0.30239 (18)	0.0670 (16)	
C4A	0.2355 (10)	0.9058 (6)	0.2585 (2)	0.084 (2)	
C5A	0.1735 (7)	0.7346 (4)	0.41484 (19)	0.0667 (17)	
C6A	0.3986 (6)	0.9188 (4)	0.47594 (15)	0.0503 (14)	
C7A	0.3940 (7)	1.0257 (4)	0.49864 (17)	0.0603 (17)	
C8A	0.4254 (7)	1.0344 (4)	0.54869 (16)	0.0633 (17)	
C9A	0.4592 (7)	0.9331 (4)	0.57459 (16)	0.0587 (14)	
C10A	0.4634 (7)	0.8257 (4)	0.55236 (16)	0.0600 (16)	
C11A	0.4341 (6)	0.8185 (4)	0.50253 (16)	0.0577 (16)	
O1B	−0.0706 (6)	0.3153 (4)	0.04016 (14)	0.0883 (16)	
O2B	0.0771 (8)	0.4308 (5)	0.40221 (15)	0.110 (2)	
O3B	0.1969 (7)	0.2600 (5)	0.40198 (14)	0.0950 (18)	
N1B	0.3210 (5)	0.4147 (4)	0.10651 (14)	0.0653 (14)	
N2B	0.3525 (5)	0.4112 (4)	0.15379 (13)	0.0637 (14)	
N3B	0.1961 (5)	0.3690 (3)	0.17486 (12)	0.0538 (11)	
N4B	0.1456 (6)	0.3479 (5)	0.38102 (15)	0.0733 (18)	
C1B	0.0636 (6)	0.3448 (4)	0.14185 (17)	0.0550 (12)	
C2B	0.1480 (6)	0.3740 (4)	0.09745 (16)	0.0550 (12)	
C3B	0.0683 (7)	0.3710 (4)	0.04782 (15)	0.0597 (16)	
C4B	0.1636 (9)	0.4365 (6)	0.00848 (19)	0.082 (2)	
C5B	−0.1210 (8)	0.3025 (5)	0.1540 (2)	0.0760 (19)	
C6B	0.1834 (6)	0.3626 (4)	0.22757 (15)	0.0533 (14)	
C7B	0.2290 (8)	0.2619 (4)	0.25147 (17)	0.0673 (16)	
C8B	0.2177 (7)	0.2566 (4)	0.30211 (19)	0.0693 (17)	
C9B	0.1605 (6)	0.3519 (5)	0.32681 (16)	0.0570 (14)	
C10B	0.1174 (7)	0.4558 (5)	0.30392 (19)	0.0703 (17)	
C11B	0.1287 (8)	0.4607 (5)	0.25360 (18)	0.0680 (17)	
H5A1	0.24880	0.66610	0.41530	0.1000*	
H7A	0.36970	1.09220	0.48030	0.0730*	
H5A2	0.06950	0.72180	0.39380	0.1000*	
H8A	0.42390	1.10620	0.56440	0.0760*	
H5A3	0.13180	0.75140	0.44750	0.1000*	
H4A1	0.17180	0.86890	0.23190	0.1260*	
H10A	0.48560	0.75900	0.57070	0.0720*	
H4A2	0.36060	0.92010	0.24920	0.1260*	
H11A	0.43810	0.74690	0.48680	0.0690*	
H4A3	0.17660	0.97820	0.26620	0.1260*	
H5B1	−0.13290	0.22300	0.14380	0.1140*	
H5B2	−0.21100	0.34910	0.13740	0.1140*	
H5B3	−0.13980	0.30770	0.18890	0.1140*	
H4B1	0.28300	0.40340	0.00310	0.1230*	
H4B2	0.17640	0.51630	0.01820	0.1230*	
H4B3	0.09310	0.43210	−0.02130	0.1230*	
H7B	0.26730	0.19720	0.23370	0.0800*	
H8B	0.24890	0.18890	0.31880	0.0830*	
H10B	0.08170	0.52040	0.32210	0.0840*	
H11B	0.10010	0.52910	0.23710	0.0820*	

### Atomic displacement parameters (Å^2^)

**Table:**

	*U*^11^	*U*^22^	*U*^33^	*U*^12^	*U*^13^	*U*^23^
O1A	0.115 (3)	0.074 (3)	0.083 (2)	−0.011 (2)	−0.018 (2)	−0.0103 (19)
O2A	0.146 (4)	0.107 (3)	0.066 (2)	0.015 (3)	−0.021 (3)	0.008 (2)
O3A	0.217 (7)	0.099 (3)	0.071 (3)	−0.001 (4)	−0.007 (3)	−0.018 (2)
N1A	0.081 (3)	0.067 (3)	0.056 (2)	−0.010 (2)	0.0006 (19)	0.0059 (19)
N2A	0.080 (3)	0.063 (2)	0.054 (2)	−0.017 (2)	0.0022 (18)	0.0112 (18)
N3A	0.0547 (19)	0.050 (2)	0.0518 (19)	−0.0050 (16)	0.0006 (14)	0.0012 (15)
N4A	0.092 (3)	0.081 (3)	0.057 (2)	−0.005 (3)	−0.003 (2)	−0.002 (2)
C1A	0.053 (2)	0.044 (2)	0.052 (2)	0.0033 (18)	−0.0018 (18)	0.0026 (17)
C2A	0.062 (2)	0.056 (3)	0.053 (2)	0.002 (2)	0.000 (2)	0.004 (2)
C3A	0.073 (3)	0.070 (3)	0.058 (2)	0.011 (3)	−0.005 (2)	−0.003 (2)
C4A	0.107 (4)	0.093 (4)	0.051 (2)	0.004 (3)	0.001 (3)	0.006 (3)
C5A	0.075 (3)	0.060 (3)	0.065 (3)	−0.009 (2)	0.002 (2)	0.007 (2)
C6A	0.051 (2)	0.055 (3)	0.045 (2)	−0.0043 (18)	0.0043 (17)	0.0041 (18)
C7A	0.066 (3)	0.055 (3)	0.060 (3)	−0.001 (2)	−0.002 (2)	0.005 (2)
C8A	0.075 (3)	0.058 (3)	0.057 (3)	−0.004 (2)	0.004 (2)	−0.006 (2)
C9A	0.059 (2)	0.069 (3)	0.048 (2)	−0.002 (2)	0.0011 (18)	0.000 (2)
C10A	0.066 (3)	0.060 (3)	0.054 (2)	−0.001 (2)	−0.001 (2)	0.007 (2)
C11A	0.064 (3)	0.052 (3)	0.057 (2)	0.002 (2)	−0.0023 (19)	0.0002 (19)
O1B	0.110 (3)	0.092 (3)	0.063 (2)	−0.039 (2)	−0.0193 (19)	0.0015 (18)
O2B	0.139 (4)	0.130 (4)	0.061 (3)	0.005 (3)	0.017 (2)	−0.022 (2)
O3B	0.105 (3)	0.123 (4)	0.057 (2)	−0.018 (3)	−0.003 (2)	0.018 (2)
N1B	0.069 (2)	0.076 (3)	0.051 (2)	−0.0069 (19)	0.0015 (18)	0.0005 (18)
N2B	0.060 (2)	0.084 (3)	0.0470 (19)	−0.012 (2)	0.0031 (16)	0.0026 (18)
N3B	0.058 (2)	0.055 (2)	0.0485 (19)	−0.0005 (17)	−0.0001 (15)	0.0000 (15)
N4B	0.074 (3)	0.099 (4)	0.047 (2)	−0.019 (3)	−0.0022 (19)	−0.004 (2)
C1B	0.062 (2)	0.050 (2)	0.053 (2)	−0.0065 (19)	0.0007 (18)	−0.0003 (18)
C2B	0.060 (2)	0.053 (2)	0.052 (2)	−0.0023 (18)	0.0045 (19)	0.0005 (18)
C3B	0.076 (3)	0.054 (3)	0.049 (2)	−0.003 (2)	−0.003 (2)	−0.0024 (19)
C4B	0.090 (4)	0.105 (5)	0.050 (2)	−0.010 (3)	0.002 (3)	0.007 (3)
C5B	0.072 (3)	0.088 (4)	0.068 (3)	−0.023 (3)	0.006 (2)	0.007 (3)
C6B	0.060 (2)	0.059 (3)	0.041 (2)	0.000 (2)	0.0024 (17)	−0.0021 (18)
C7B	0.090 (3)	0.059 (3)	0.053 (2)	0.009 (2)	0.004 (2)	0.004 (2)
C8B	0.084 (3)	0.065 (3)	0.059 (3)	0.006 (3)	0.004 (2)	0.011 (2)
C9B	0.055 (2)	0.072 (3)	0.044 (2)	−0.010 (2)	0.0004 (17)	0.000 (2)
C10B	0.082 (3)	0.070 (3)	0.059 (3)	0.005 (3)	0.003 (2)	−0.012 (2)
C11B	0.089 (3)	0.060 (3)	0.055 (3)	0.009 (3)	0.002 (2)	0.000 (2)

### Geometric parameters (Å, º)

**Table:**

O1A—C3A	1.211 (7)	C4A—H4A2	0.9600
O2A—N4A	1.206 (7)	C4A—H4A3	0.9600
O3A—N4A	1.213 (7)	C5A—H5A3	0.9600
O1B—C3B	1.215 (7)	C5A—H5A2	0.9600
O2B—N4B	1.221 (8)	C5A—H5A1	0.9600
O3B—N4B	1.220 (8)	C7A—H7A	0.9300
N1A—N2A	1.295 (6)	C8A—H8A	0.9300
N1A—C2A	1.368 (6)	C10A—H10A	0.9300
N2A—N3A	1.374 (6)	C11A—H11A	0.9300
N3A—C1A	1.352 (6)	C1B—C2B	1.397 (6)
N3A—C6A	1.428 (5)	C1B—C5B	1.467 (7)
N4A—C9A	1.464 (6)	C2B—C3B	1.471 (6)
N1B—N2B	1.308 (5)	C3B—C4B	1.482 (7)
N1B—C2B	1.366 (6)	C6B—C7B	1.370 (6)
N2B—N3B	1.364 (5)	C6B—C11B	1.391 (7)
N3B—C1B	1.347 (6)	C7B—C8B	1.382 (7)
N3B—C6B	1.440 (5)	C8B—C9B	1.352 (7)
N4B—C9B	1.480 (6)	C9B—C10B	1.384 (8)
C1A—C5A	1.468 (7)	C10B—C11B	1.373 (7)
C1A—C2A	1.390 (6)	C4B—H4B1	0.9600
C2A—C3A	1.466 (7)	C4B—H4B2	0.9600
C3A—C4A	1.488 (8)	C4B—H4B3	0.9600
C6A—C7A	1.377 (6)	C5B—H5B1	0.9600
C6A—C11A	1.387 (6)	C5B—H5B2	0.9600
C7A—C8A	1.385 (6)	C5B—H5B3	0.9600
C8A—C9A	1.384 (6)	C7B—H7B	0.9300
C9A—C10A	1.376 (6)	C8B—H8B	0.9300
C10A—C11A	1.376 (6)	C10B—H10B	0.9300
C4A—H4A1	0.9600	C11B—H11B	0.9300
			
N2A—N1A—C2A	109.4 (4)	C8A—C7A—H7A	120.00
N1A—N2A—N3A	107.1 (4)	C6A—C7A—H7A	120.00
N2A—N3A—C1A	111.3 (3)	C9A—C8A—H8A	121.00
N2A—N3A—C6A	117.5 (3)	C7A—C8A—H8A	121.00
C1A—N3A—C6A	131.2 (4)	C9A—C10A—H10A	121.00
O2A—N4A—O3A	122.3 (5)	C11A—C10A—H10A	120.00
O2A—N4A—C9A	119.1 (4)	C6A—C11A—H11A	120.00
O3A—N4A—C9A	118.7 (5)	C10A—C11A—H11A	120.00
N2B—N1B—C2B	109.2 (4)	N3B—C1B—C2B	102.3 (4)
N1B—N2B—N3B	106.2 (3)	N3B—C1B—C5B	125.0 (4)
N2B—N3B—C1B	113.0 (3)	C2B—C1B—C5B	132.7 (4)
N2B—N3B—C6B	119.4 (3)	N1B—C2B—C1B	109.4 (4)
C1B—N3B—C6B	127.5 (4)	N1B—C2B—C3B	122.5 (4)
O3B—N4B—C9B	118.0 (5)	C1B—C2B—C3B	128.0 (4)
O2B—N4B—O3B	123.5 (4)	O1B—C3B—C2B	119.9 (4)
O2B—N4B—C9B	118.5 (5)	O1B—C3B—C4B	122.3 (4)
N3A—C1A—C5A	125.6 (4)	C2B—C3B—C4B	117.9 (4)
C2A—C1A—C5A	130.7 (4)	N3B—C6B—C7B	120.1 (4)
N3A—C1A—C2A	103.4 (4)	N3B—C6B—C11B	119.0 (4)
N1A—C2A—C1A	108.9 (4)	C7B—C6B—C11B	120.9 (4)
N1A—C2A—C3A	121.9 (4)	C6B—C7B—C8B	119.8 (4)
C1A—C2A—C3A	129.1 (4)	C7B—C8B—C9B	118.6 (4)
O1A—C3A—C2A	121.1 (5)	N4B—C9B—C8B	119.5 (5)
O1A—C3A—C4A	121.0 (5)	N4B—C9B—C10B	117.3 (5)
C2A—C3A—C4A	117.8 (5)	C8B—C9B—C10B	123.1 (4)
N3A—C6A—C11A	119.9 (4)	C9B—C10B—C11B	118.1 (5)
N3A—C6A—C7A	119.2 (4)	C6B—C11B—C10B	119.5 (5)
C7A—C6A—C11A	120.9 (4)	C3B—C4B—H4B1	109.00
C6A—C7A—C8A	120.1 (4)	C3B—C4B—H4B2	109.00
C7A—C8A—C9A	118.0 (4)	C3B—C4B—H4B3	109.00
C8A—C9A—C10A	122.4 (4)	H4B1—C4B—H4B2	109.00
N4A—C9A—C8A	118.9 (4)	H4B1—C4B—H4B3	110.00
N4A—C9A—C10A	118.7 (4)	H4B2—C4B—H4B3	110.00
C9A—C10A—C11A	119.0 (4)	C1B—C5B—H5B1	109.00
C6A—C11A—C10A	119.6 (4)	C1B—C5B—H5B2	109.00
C3A—C4A—H4A1	109.00	C1B—C5B—H5B3	109.00
C3A—C4A—H4A2	109.00	H5B1—C5B—H5B2	110.00
H4A1—C4A—H4A2	110.00	H5B1—C5B—H5B3	109.00
H4A1—C4A—H4A3	109.00	H5B2—C5B—H5B3	109.00
C3A—C4A—H4A3	109.00	C6B—C7B—H7B	120.00
H4A2—C4A—H4A3	109.00	C8B—C7B—H7B	120.00
C1A—C5A—H5A1	109.00	C7B—C8B—H8B	121.00
C1A—C5A—H5A2	109.00	C9B—C8B—H8B	121.00
C1A—C5A—H5A3	109.00	C9B—C10B—H10B	121.00
H5A1—C5A—H5A2	109.00	C11B—C10B—H10B	121.00
H5A1—C5A—H5A3	110.00	C6B—C11B—H11B	120.00
H5A2—C5A—H5A3	110.00	C10B—C11B—H11B	120.00
			
C2A—N1A—N2A—N3A	−0.3 (5)	N3A—C1A—C2A—C3A	−177.7 (5)
N2A—N1A—C2A—C1A	0.3 (5)	C5A—C1A—C2A—C3A	−3.4 (8)
N2A—N1A—C2A—C3A	178.0 (4)	C5A—C1A—C2A—N1A	174.2 (5)
N1A—N2A—N3A—C1A	0.2 (5)	C1A—C2A—C3A—O1A	−14.0 (8)
N1A—N2A—N3A—C6A	−180.0 (4)	C1A—C2A—C3A—C4A	164.8 (5)
N2A—N3A—C1A—C2A	0.0 (5)	N1A—C2A—C3A—C4A	−12.5 (7)
N2A—N3A—C1A—C5A	−174.7 (4)	N1A—C2A—C3A—O1A	168.8 (5)
C6A—N3A—C1A—C2A	−179.9 (4)	N3A—C6A—C11A—C10A	179.1 (4)
C6A—N3A—C1A—C5A	5.4 (7)	N3A—C6A—C7A—C8A	−178.3 (4)
N2A—N3A—C6A—C7A	37.8 (6)	C11A—C6A—C7A—C8A	0.1 (7)
N2A—N3A—C6A—C11A	−140.7 (4)	C7A—C6A—C11A—C10A	0.6 (7)
C1A—N3A—C6A—C7A	−142.4 (5)	C6A—C7A—C8A—C9A	−0.6 (7)
C1A—N3A—C6A—C11A	39.1 (7)	C7A—C8A—C9A—C10A	0.3 (8)
O2A—N4A—C9A—C8A	−171.7 (5)	C7A—C8A—C9A—N4A	−179.5 (5)
O2A—N4A—C9A—C10A	8.5 (8)	N4A—C9A—C10A—C11A	−179.7 (4)
O3A—N4A—C9A—C8A	8.2 (8)	C8A—C9A—C10A—C11A	0.4 (8)
O3A—N4A—C9A—C10A	−171.7 (6)	C9A—C10A—C11A—C6A	−0.9 (7)
C2B—N1B—N2B—N3B	−0.7 (5)	N3B—C1B—C2B—N1B	−0.9 (5)
N2B—N1B—C2B—C1B	1.1 (6)	N3B—C1B—C2B—C3B	−177.2 (4)
N2B—N1B—C2B—C3B	177.6 (4)	C5B—C1B—C2B—N1B	177.0 (5)
N1B—N2B—N3B—C6B	−175.6 (4)	C5B—C1B—C2B—C3B	0.7 (9)
N1B—N2B—N3B—C1B	0.2 (5)	N1B—C2B—C3B—O1B	166.6 (5)
N2B—N3B—C1B—C2B	0.5 (5)	N1B—C2B—C3B—C4B	−12.9 (7)
N2B—N3B—C1B—C5B	−177.6 (5)	C1B—C2B—C3B—O1B	−17.6 (8)
C1B—N3B—C6B—C11B	−90.7 (6)	C1B—C2B—C3B—C4B	162.9 (5)
C6B—N3B—C1B—C5B	−2.3 (7)	N3B—C6B—C7B—C8B	179.6 (4)
N2B—N3B—C6B—C7B	−94.0 (5)	C11B—C6B—C7B—C8B	1.1 (8)
C6B—N3B—C1B—C2B	175.9 (4)	N3B—C6B—C11B—C10B	−179.5 (5)
C1B—N3B—C6B—C7B	90.9 (6)	C7B—C6B—C11B—C10B	−1.1 (8)
N2B—N3B—C6B—C11B	84.5 (5)	C6B—C7B—C8B—C9B	0.3 (8)
O3B—N4B—C9B—C8B	4.5 (7)	C7B—C8B—C9B—N4B	179.4 (5)
O2B—N4B—C9B—C10B	8.1 (7)	C7B—C8B—C9B—C10B	−1.8 (8)
O2B—N4B—C9B—C8B	−173.1 (5)	N4B—C9B—C10B—C11B	−179.4 (5)
O3B—N4B—C9B—C10B	−174.3 (5)	C8B—C9B—C10B—C11B	1.9 (8)
N3A—C1A—C2A—N1A	−0.2 (5)	C9B—C10B—C11B—C6B	−0.4 (8)

### Hydrogen-bond geometry (Å, º)

**Table:**

*D*—H···*A*	*D*—H	H···*A*	*D*···*A*	*D*—H···*A*
C5*B*—H5*B*1···O3*A*^i^	0.96	2.51	3.306 (8)	141
C10*B*—H10*B*···O1*A*	0.93	2.58	3.197 (7)	124

Symmetry code: (i) −*x*+1/2, *y*−1, *z*−1/2.
